# Robotics in Cardiac Surgery: Past, Present, and Future

**DOI:** 10.5041/RMMJ.10117

**Published:** 2013-07-25

**Authors:** Bryan Bush, L. Wiley Nifong, W. Randolph Chitwood

**Affiliations:** 1 Division of Cardiothoracic Surgery, Department of Cardiovascular Sciences, East Carolina University, Brody School of Medicine, Greenville, North Carolina, USA;; 2 Associate Professor of Surgery, Department of Cardiovascular Sciences, East Carolina University, Brody School of Medicine, Director, Center for Robotics and Minimally Invasive Surgery, East Carolina University, Brody School of Medicine, Greenville, North Carolina, USA;; 3 Director, East Carolina Heart Institute, Professor of Cardiovascular Surgery, East Carolina University, Brody School of Medicine, Greenville, North Carolina, USA

**Keywords:** Cardiac surgery, minimally invasive, robotics, surgical procedures, thoracic surgery

## Abstract

Robotic cardiac operations evolved from minimally invasive operations and offer similar theoretical benefits, including less pain, shorter length of stay, improved cosmesis, and quicker return to preoperative level of functional activity. The additional benefits offered by robotic surgical systems include improved dexterity and degrees of freedom, tremor-free movements, ambidexterity, and the avoidance of the fulcrum effect that is intrinsic when using long-shaft endoscopic instruments. Also, optics and operative visualization are vastly improved compared with direct vision and traditional videoscopes. Robotic systems have been utilized successfully to perform complex mitral valve repairs, coronary revascularization, atrial fibrillation ablation, intracardiac tumor resections, atrial septal defect closures, and left ventricular lead implantation. The history and evolution of these procedures, as well as the present status and future directions of robotic cardiac surgery, are presented in this review.

## INTRODUCTION

The purported benefits of minimally invasive cardiac surgery have been well described; smaller, less invasive incisions have the theoretical benefit of less pain, shorter length of stay, improved cosmesis, and quicker return to preoperative level of functional activity. Minimally invasive approaches have become the standard of care at many institutions, and excellent results have been achieved.

As minimally invasive cardiac operations gained favor, developments in tele-manipulation technology and optics fostered the evolution of robotic-assisted cardiac surgery. Currently, the da Vinci™ surgical system (Intuitive Surgical, Sunnyvale, CA, USA) is the only US Food and Drug Administration (FDA)-approved robotic system used for cardiac surgical procedures. Today, robotic heart surgeons perform complex mitral valve repairs, coronary revascularizations, atrial fibrillation ablations, intracardiac tumor resections, and congenital heart surgery procedures.

Before robotic cardiac surgery became a viable technique, minimally invasive heart surgery had been developed and perfected. These alternatives to the gold standard median sternotomy incision became increasingly commonplace as advances in minimally invasive access and exposure, perfusion strategies, and myocardial protection were made. The development of these techniques enabled the application of endoscopic visualization and instrumentation to be developed. This advancing pathway led to the robotic tele-manipulation of materials and tissues that we have today. The da Vinci™ surgical system provides increased operative dexterity for surgeons. The wrist-like articulating instruments move with six degrees of freedom, compared with the four degrees of freedom that endoscopic instruments provide. Other benefits are tremor-free movements, ambidexterity, and the avoidance of the fulcrum effect that is intrinsic when using long-shaft endoscopic instruments. Moreover, the system improves operative visualization greatly through the use of three-dimensional high-definition imaging.

## MITRAL VALVE SURGERY

The most commonly performed robot-assisted cardiac procedure today is a mitral valve repair or replacement. As in other less invasive cardiac operations, minimally invasive and subsequently robotic mitral valve surgery evolved from modifications of incisions performed previously under direct vision. Large series from Cohn and Cosgrove showed that mitral surgery, done via minimal access incisions and performed under direct vision, offered comparable results to the sternotomy approach (mortality 1%–3%).^1^,^2^ The next step forward was to perform mitral surgery using videoscopic assistance. The first mitral repair using a videoscope was performed by Carpentier in 1996,^3^ and the first mitral valve replacement was done by Chitwood later the same year.^4^ The Leipzig Heart Center experience was reported by Mohr in 1998 and showed excellent results in 51 patients who underwent simple mitral repair or replacement operations.^5^ At the same meeting, Chitwood reported a 30-day operative mortality of 3.2% with no major complications in 31 patients. This series consisted of a variety of complex repairs, including quadrangular resections, sliding valvuloplasties, and chordal replacements.^6^

The first robotic mitral repair was performed by Carpentier in 1998, using an early prototype of the da Vinci™ surgical system.^7^ The following week, Mohr repaired five mitral valves and performed a coronary revascularization with the device.^8^ The first robotic mitral repair in North America was performed by Chitwood in 2000, and consisted of a large P_2_ trapezoidal resection with an intracorporeal suture repair followed by annuloplasty band implantation.^9^ Two subsequent FDA investigational device clinical trials led to approval in 2002 of the da Vinci™ surgical system for mitral valve surgery in the United States.^10^,^11^

Mihaljevic et al. reported their results for 261 robotic mitral valve repairs done between 2006 and 2009.^12^ Their results were compared with mitral valve repairs done via complete sternotomy (*n* = 114), partial sternotomy (*n* = 270), and right mini-anterolateral thoracotomy (*n* = 114). Outcomes were compared on an intent-to-treat basis using propensity score matching. Median cardiopulmonary bypass time was 42 minutes longer for robotic than for complete sternotomy, 39 minutes longer than partial sternotomy, and 11 minutes longer than right mini-anterolateral thoracotomy (*P < 0.*0001). There were no in-hospital deaths in any group, and neurologic, pulmonary, and renal complications were similar among groups (*P* > 0.1). The robotic group had the lowest occurrences of atrial fibrillation and pleural effusion, contributing to the shortest hospital stay (median 4.2 days); 1.0, 1.6, and 0.9 days shorter than for complete sternotomy, partial sternotomy, and right mini-anterolateral thoracotomy (all *P <* 0.001), respectively.

Similar reductions in length of stay were seen at the University of Pennsylvania in a comparison of 39 patients who underwent sternotomy and mitral valve repair, or replacement, with 26 patients who underwent robotically assisted mitral valve repair or replacement.^13^ Patients who underwent robotic-assisted surgery experienced shorter mean duration of postoperative hospitalization (7.1 versus 10.6 days; *P* = 0.04), despite longer cross-clamp and bypass times (110 versus 151 minutes, *P* = 0.0015; 162 versus 239 minutes, *P* = 0.001, respectively). Mean packed red blood cell transfusion was also lower among patients who underwent robotic-assisted mitral valve surgery (5.0 versus 2.8 units, *P* = 0.04).

Today, most robot-assisted mitral valve repairs are accomplished either through a 3–4-cm right anterolateral mini-thoracotomy or a 2-cm lateral working port. The articulating EndoWrist™ (Intuitive Surgical, Sunnyvale, CA, USA) instruments and dynamic left atrial retractor allow console surgeons to employ Carpentier’s and others’ “toolbox” of repair techniques.

Our institution has performed over 800 robotic mitral valve repairs. Results have been published for the first 540 patients.^14^ Of these, 454 patients underwent a lone mitral repair, and 86 had a concomitant atrial fibrillation ablation. The average cardiopulmonary bypass and cross-clamp times were 153 and 116 minutes, respectively, in the lone mitral repair patients. The group operative mortality was 0.4%. The mean follow-up period was 351 days (15–946 days), and 2.9% of patients required a reoperation for a failed repair. The cardiopulmonary bypass and arrest times have improved with on-going experience. In the first FDA trial, the average cross-clamp time was 150 minutes.^10^ In the second multicenter FDA trial, the average cross-clamp time fell to 126 minutes, and there was little variation in operative time between centers.^11^

We use topographic valve models, derived from intra-operative high-quality three-dimensional transesophageal echocardiography images to plan a successful repair. To isolate the right lung, patients are either intubated with a dual-lumen endotracheal tube or single-lumen tube with bronchial blocker. The patient’s right side then is elevated to 30 degrees. Cardiopulmonary bypass is achieved via bicaval venous cannulation (right internal jugular and femoral veins) and femoral arterial cannulation. In patients with either inadequate femoral artery size or aorto-iliac atherosclerotic disease, the right axillary artery is cannulated through an 8-mm polytetrafluoroethylene (PTFE) side-arm graft. The aorta is occluded using the Chitwood transthoracic aortic cross-clamp (Scanlan International, Minneapolis, MN, USA), and antegrade crystalloid Bretschneider’s cold cardioplegia is used to arrest the heart. In reoperative cases and patients with an atherosclerotic or calcified ascending aorta, hypothermic (26°C) fibrillatory arrest is used for myocardial protection. Thereafter, robotic instrument arm trocars are inserted into the chest, and the da Vinci™ surgical cart is docked by the patient’s left side.^14^ Most commonly we use the following techniques to perform complex mitral repairs: 1) limited triangular or quadrangular resection, 2) folding valvuloplasty, 3) chordal shortening either by translocation or papillary muscle folding, 4) neochord implantation, and rarely 5) a leaflet sliding-plasty. Formerly we tied all suture knots intracorporeally; however, we now use the Cor-Knot™ suture device (LSI Solutions, Victor, NY, USA), to secure annuloplasty bands. Implementation of this device into our routine has significantly reduced our cardiopulmonary bypass and cross-clamp times.^15^

## CORONARY REVASCULARIZATION

The da Vinci™ surgical system has been used very successfully to harvest the internal thoracic artery (ITA) for coronary artery bypass grafting (CABG). In most cases the ITA-coronary anastomosis has been hand-sewn via either a mini-thoracotomy or median sternotomy. However, several surgeons have shown good results on both beating and arrested hearts with totally endoscopic robotic coronary artery bypass grafting (TECAB).

Using a first-generation da Vinci™ surgical system, the first TECAB was performed in two patients by Loulmet et al. in 1998.^16^

Srivastava et al. reported results from 150 patients who underwent a robotic ITA harvest with off-pump CABG via a mini-thoracotomy.^17^ Later, two patients presented with symptomatic graft occlusion and were treated successfully by a percutaneous intervention, and all grafts were patent in 55 patients by computed tomographic angiography at three months.

Argenziano reported the FDA multicenter robotic coronary bypass Investigational Device Exemption trial in 2006.^18^ Ninety-eight patients who required a single-vessel left anterior descending (LAD) revascularization were enrolled at 12 centers. Of these, 13 patients were excluded intra-operatively for various reasons. Of the 85 remaining patients who underwent a TECAB, there were 6% conversions to an open sternotomy, no deaths, no strokes, one early re-intervention, and one myocardial infarction. Coronary angiography at three months revealed greater than a 50% stenosis or an occlusion in 7.1% of patients.

The largest TECAB experience to date was published by Bonaros et al.^19^ Five hundred patients from two institutions underwent either a single (*n* = 334), double (*n* = 150), triple (*n* = 15), or quadruple (*n* = 1) bypass. One-third of the cases had a hybrid procedure, i.e. CABG combined with percutaneous coronary intervention. The median operative, cardiopulmonary bypass, and cross-clamp times were 305 minutes, 98 minutes, and 73 minutes, respectively. Bilateral internal thoracic arteries were used in 22% of patients with a median harvest time of 34 minutes for the left and 32 minutes for the right. The operative mortality was 1% with 10% having conversions to a sternotomy and 5% having ITA harvest injuries. Major morbidity and mortality was 5% as defined by death, myocardial infarction, stroke, vascular complication, or long-term ventilation requiring a tracheostomy. Operative success, as defined by freedom from repeat revascularization, reoperation for bleeding, or conversion to a larger incision, was present in 80% of patients.

The same group reported on their length of stay results for 541 consecutive TECAB patients in two different institutions on different continents.^20^ Their overall observed median length of stay (LOS) was 6 days (range 2–54 days, mean 7.35 days). These data are slightly better than LOS data reported by Swaminathan and colleagues for CABG patients treated during a 17-year period using the Nationwide Inpatient Sample (NIS) database, which contains information relating to all inpatients of non-federal hospitals across the United States.^21^ In this report, median LOS among 8,398,554 CABG discharges decreased from 11 to 8 days between 1988 and 2005 (*P* < 0.0001). In a more recent cohort, the SYNTAX trial, which compared multivessel drug-eluting stenting with multivessel CABG in patients with triple-vessel or left main coronary disease during the 2005 to 2007, reported a postoperative LOS in the CABG cohort (*n* = 897) of 9.5 ± 8 days.^22^

The operative approach, as described by Bonaros et al., was as follows.^19^ Suitability for arrested heart TECAB was determined by preoperative CT angiography. Patients with aortic or peripheral atherosclerosis were scheduled for beating heart TECAB. The authors preferred an arrested heart approach giving better-quality control over performing coronary anastomoses. Three robotic arm trocars, one camera port and two working ports, were introduced into the left (or, if the right coronary artery was grafted, into the right) hemithorax under single-lung ventilation and carbon dioxide insufflation (6 to 10 mmHg). In arrested heart TECAB procedures, the femoral or axillary artery was cannulated, and an aortic endo-occlusion balloon catheter (Endo CPB, Edwards Lifesciences, Irvine, CA, or Estech, San Ramon, CA, USA) was used to occlude the ascending aorta. The femoral vein was also cannulated. In off-pump TECABs, peripheral cannulation via the femoral or axillary artery and femoral vein were established in order to be prepared to institute cardiopulmonary bypass in the event of hemodynamic instability, myocardial ischemia, or significant space limitations inside the chest. The internal thoracic artery was then harvested, in a skeletonized fashion, in order to optimize graft length. After resection of the pericardial fat pad and a pericardiotomy, the coronary anastomoses were performed in a hand-sewn running fashion using 7-0 polypropylene suture. In multivessel TECABs, the lateral and back walls of the heart were exposed using an endoscopic suction stabilizer. An additional 5-mm port in the fourth intercostal space left parasternally allowed the patient side surgeon to aid in exposure. Intra-operative assessment by graft angiography or Doppler flow measurement was performed.

To summarize, these reports have shown some success with robot-assisted revascularization in properly selected patients. However, it is clear that TECAB is a challenging procedure, and the learning curve is significant. The limited data on this subject suggest that robotic coronary operations still have a long way to go to uniformly have the same results as traditional coronary grafting. Moreover, committed and large clinical volume robotic coronary surgeons have obtained the best results. The newest generation of the da Vinci™ SI robotic system with a fourth arm and an endostabilizer may enable more complex bypass operations to be done on both beating and arrested hearts. At several centers, and with increasing frequency, TECAB is being done in concert with a percutaneous coronary intervention as a hybrid operation. This combines the survival benefits of left internal thoracic artery (LITA) to LAD grafting with the benefits of minimally invasive access of the percutaneous coronary intervention (PCI).

## ATRIAL FIBRILLATION ABLATION

The excellent results using the classic Coxmaze for treating atrial fibrillation operation have been well documented.^23^ Given the known failure rate of medical therapy and catheter-based ablation, as well as the prevalence of atrial fibrillation in the general population (1%–2%) and the elderly (9% in patients over 80), the appeal of minimally invasive atrial fibrillation surgery is obvious.^24^ Similar to other cardiac operations, the da Vinci™ surgical system offers the benefit of improved dexterity and outstanding visualization, making it an ideal device for the precise endocardial placement of probes for atrial fibrillation ablation.

Despite a lack of level I evidence, cryoablation is gaining traction among surgeons as a safe and effective treatment option for atrial fibrillation. Several small retrospective, randomized clinical trials have shown a conversion rate to sinus rhythm at 12 months in the range of 60%–80%.^25^

In 2012, we reported a series of 86 patients who underwent combined robotic mitral valve repair with concomitant cryomaze.^14^ Our technique, as previously described,^26^ consists of positioning an 8-cm flexible argon-cooled flexible probe (Medtronic, Minneapolis, MN, USA) to reproduce the Coxmaze III lesion set. Specialized robotic forceps are used to grasp and position the probe. Freedom from atrial fibrillation was seen in 83 patients (96.5%) at a follow-up period of 351 ± 281 days. Cardiopulmonary bypass times were longer when cryoablation was added to lone mitral valve repair (189 minutes versus 153 minutes). Cross-clamp times were also longer (131 minutes versus 117 minutes). Although longer-term follow-up and level I evidence are lacking, we believe that cryoablation is a safe and effective technique for the treatment of atrial fibrillation. The robotically assisted, right mini-thoracotomy approach may prove to be an ideal minimally invasive surgical treatment for atrial fibrillation, whether combined with mitral valve surgery or done as a stand-alone operation.

Robotically assisted epicardial ablation with microwave energy has also been performed with few complications. The largest series, by Pruitt and colleagues, reported on 33 paroxysmal and 17 permanent atrial fibrillation patients who underwent thoracoscopic or robotic-assisted off-pump epicardial microwave ablation. The investigators reported no perioperative death, a mean length of stay of 4 days, and a 79.5% success rate overall, with a cure rate of 93.5% in paroxysmal disease versus 69.2% in permanent disease.^27^

## OTHER CARDIAC PROCEDURES

Epicardial left ventricular (LV) lead insertion for cardiac resynchronization therapy offers an important rescue therapy for failed percutaneous coronary sinus LV lead placement. Robot-assisted LV lead placement is an enticing and safe alternative to more invasive epicardial lead surgery in this very-high-risk population of patients with poor ventricular function. Often the enlarged ventricle in these patients presents a technical challenge, which can be safely overcome using robotic assistance. Kamath et al. reported 78 consecutive patients, who underwent a robot-assisted epicardial lead placement, and found improvement in both pacing thresholds and lead impedance over both the short (<12 months) and long term (>12 months). At 44 ± 21 months’ follow-up there were 20 deaths (26%). These patients were older (77 ± 7 versus 67 ± 11 years, *P* = 0.001) and had a lower ejection fraction (13% ± 7% versus 18% ± 9%, *P* = 0.02) than surviving patients.^28^ In 2006, Derose et al. published their midterm results from 42 patients who underwent robot-assisted LV replacement. There were no mortalities or technical failures.^29^

Although much less common than mitral valve surgery, coronary revascularization, or atrial fibrillation ablation, several case reports exist in the literature for other cardiac procedures, such as intracardiac tumor resections and atrial septal defect (ASD) closures. Murphy et al. reported three successful atrial myxoma resections using the da Vinci™ surgical system in 2005.^30^ In 2012, Schilling et al. reported their results for 17 robot-assisted atrial myxoma resections using the da Vinci™ surgical system between 2000 and 2009.^31^ Despite no difference in perfusion or cross-clamp times, total operative time was shorter in the robot-assisted group as compared with 29 patients who underwent traditional myxoma resection during the same time period (2.7 hours versus 3.5 hours, *P* = 0.02). There were no mortalities, reoperations for bleeding, strokes, or wound infections in either group. There was no significant difference in administration of blood products, incidence of pneumonia, renal failure, or atrial fibrillation. Additionally, there was no difference in length of hospital stay or intensive care unit length of stay between the robotic-assisted and traditional myxoma surgical procedure groups.

Torracca et al. and Wimmer-Greinecker et al. were the first to report small series of patients undergoing robotic atrial septal defect (ASD) closures.^32^,^33^ In a FDA Investigational Device Exemption trial, Argenziano et al. demonstrated that ASD closure in adults can be performed safely and effectively using the da Vinci™ surgical system.^34^ Their median cross-clamp time was 32 minutes. Bonaros et al. demonstrated no mortalities or residual shunts in 17 patients undergoing robot-assisted ASD closures.^35^ This study demonstrated a steep operative time learning curve. Gao et al. reported on 24 patients who underwent robot-assisted ASD closures with the heart beating;14 of these defects were repaired with an autologous pericardial patch, and 10 were closed primarily. There were no mortalities or residual ASD by echocardiography.^36^ As many of these procedures were done in young, physically active patients, the robotically assisted, totally endoscopic approach of ASD closure offers the tangible benefits of decreased pain, sternal stability, and improved cosmesis.

## THE FUTURE

Currently, there are several successful robotic cardiac surgery centers. Whether this technology will continue to gain more widespread acceptance remains to be seen. Despite all of the above-described benefits, several limitations have hampered the acceptance of robotic heart surgery. With improved technology, many of these limitations should also diminish. For instance, many surgeons remain concerned about the lack of haptic feedback. Robotic surgeons have become familiar with “ocular tactility,” relying on visual tissue deformation to judge the amount of force being applied to tissues. In our experience the lack of haptic feedback has not been a concern. Future robotic systems will likely incorporate strain sensors to the instrument arms, allowing for haptic feedback and precise control of force. Instrument and camera sizes will decrease, and optics will improve, allowing for smaller incisions. A greater variety of robotic instruments will be developed, allowing for more operative options and improved dexterity. Advances in three-dimensional echocardiography and modeling software will continue to be made, possibly allowing a “blueprint” model to be overlaid on the operative field image at the console.

It has become evident that in order to achieve success with a robotic cardiac surgery program, several key elements are required. For one, the concept of a highly specialized and trained robotic team is paramount, to include anesthesiologists, perfusionists, operating room staff, nurses, and surgeons. With limited access to and visualization of the heart, skilled echocardiographers are crucial. Achieving safe cannulation, planning for complex valve repairs, and monitoring cardiac function are all predicated on high-quality, three-dimensional transesophageal echocardiography. Finally, robotic heart surgery centers must have an adequate referral base to attain safety and efficiency. To date, several centers have achieved success in robotic cardiac surgery, performing a variety of heart operations reproducibly, reliably, effectively, and safely. We are confident that this promising technology will continue to advance and grow in utilization internationally.

## Abbreviations

ASD: atrial septal defect;
CABG: coronary artery bypass graft;
FDA: Food and Drug Administration;
ITA: internal thoracic artery;
LAD: left anterior descending;
LOS: length of stay;
LV: left ventricular;
PCI: percutaneous coronary intervention;
PTFE: polytetrafluoroethylene;
TECAB: totally endoscopic coronary artery bypass.
